# Changes in Medicare Part B Spending for Biologic Drugs After Biosimilar Entry Into the Market

**DOI:** 10.1001/jamahealthforum.2021.2634

**Published:** 2021-09-17

**Authors:** Emma Boswell Dean, Amelia M. Bond

**Affiliations:** 1 Department of Health Management and Policy, Miami Herbert Business School, University of Miami, Miami, Florida; 2 Division of Health Policy and Economics, Department of Population Health Sciences, Weill Cornell Medical College, New York, New York

## Abstract

This cross-sectional study describes changes in annual Medicare Part B spending for biologic drugs after biosimilar entry, focusing on the first 4 products to experience biosimilar competition: filgrastim, infliximab, epoetin alpha, and pegfilgrastim.

## Introduction

Biosimilars have the potential to reduce Medicare drug spending.^1,2^ Biosimilars are genericlike alternatives to biologic drugs—complex medicines derived from biological sources—that currently account for 37% of US prescription drug spending.^3^ In the retail pharmacy sector, the entry of generic medicines leads to quick increases in generic market share and considerable decreases in total spending; however, savings from initial biosimilar launches have been smaller than predicted.^4^ In this cross-sectional study, we describe and decompose changes in annual Medicare Part B spending for biologic drugs after biosimilar entry, focusing on the first 4 products to experience biosimilar competition: filgrastim, infliximab, epoetin alfa, and pegfilgrastim.

## Methods

We obtained total annual administration volume (allowed services) and Medicare spending (payment) for 2013 through 2019 from the Centers for Medicare & Medicaid Services’ Part B National Summary Data Files. For each product, we converted administration volume to a consistent dose (filgrastim, 1 μg; infliximab, 10 mg; epoetin alfa, 1000 units; and pegfilgrastim, 6 mg) and calculated observed prices by dividing Medicare spending by the standardized volume. For each product class, we calculated the biosimilar market share based on standardized administration volume.

We graphed annual originator and biosimilar prices and biosimilar market share by product class. Next, we calculated annual total spending change by product class and decomposed this into changes in originator and biosimilar observed prices, biosimilar market share, and overall volume using the Congressional Budget Office’s decomposition method of physician service spending (eMethods in the Supplement).^5^ No statistical tests were used because the Part B National Summary Data Files use all Medicare Part B claims.

This study was deemed exempt by the institutional review board at Weill Cornell Medical College. We followed the Strengthening the Reporting of Observational Studies in Epidemiology (STROBE) reporting guidelines for cross-sectional studies.

## Results

Between 2013 and a product’s first biosimilar launch, originator prices rose 20%, 52%, and 17% for infliximab, pegfilgrastim, and epoetin alfa, respectively, but were stable for filgrastim (Figure). After biosimilar launch, originator filgrastim and pegfilgrastim prices remained stable, while originator prices declined for infliximab and epoetin alfa. When they launched, biosimilar prices entered at levels comparable with originator prices, then declined by 37%, 24%, 6%, and 11% for filgrastim, infliximab, pegfilgrastim, and epoetin alfa, respectively, by 2019. Biosimilar market share by the end of 2019 varied substantially across product categories; filgrastim, infliximab, pegfilgrastim, and epoetin alfa biosimilar market share were 75%, 9%, 17%, and 19%, respectively.

**Figure.**
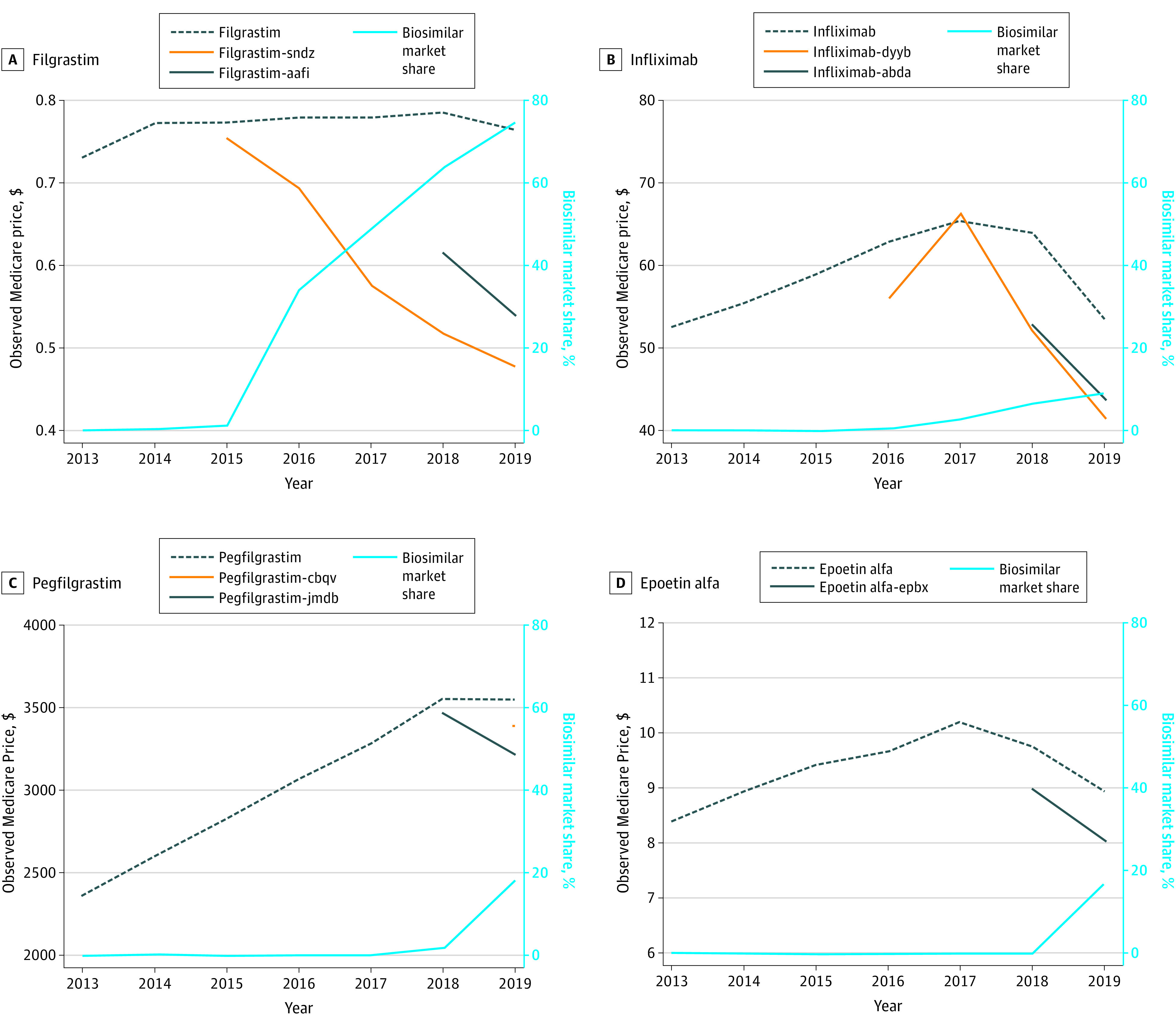
Observed Medicare Price and Biosimilar Market Share, 2013-2019 Observed Medicare payment represents the average payment or price a health care professional received for a consistent dose of a given product (filgrastim, 1 μg; infliximab, 10 mg; epoetin alfa, 1000 units; and pegfilgrastim, 6 mg). The price includes Medicare payment as well as co-payment. Observed Medicare payment and biosimilar market share were calculated from the Centers for Medicare & Medicaid Services’ Part B National Summary Data Files.

The Table^5^ summarizes changes in total spending. Prior to biosimilar launch, filgrastim annual total spending declined by 9%, driven primarily by volume declines, while changes in annual total spending for infliximab, pegfilgrastim, and epoetin alfa were nominal because volume declines were offset by price increases. After biosimilar launch, total spending declined annually in all product categories (average annual decline was 13% for filgrastim and epoetin alfa, 7% for infliximab, and 1% for pegfilgrastim). About half of the declines were driven by volume for epoetin alfa and filgrastim; other declines corresponded to pricing and market share trends (Figure).

**Table.** Decomposition of Annual Change in Total Medicare Part B Spending by Product, 2013-2019^a^

Product	Years	Spend in baseline year, $	Total change in spending (%), $	Change in spending (%), $			
				Total volume (originator + biosimilar units)	Biosimilar market share	Originator price	Biosimilar prices
Filgrastim	2013-2014	75 556 952	−7 445 440 (−10)	−12 369 532 (166)	NA	4 924 089 (−66)	NA
	2014-2015	68 111 512	−6 065 096 (−9)	−6 094 098 (100)	−14 512 (0)	43 524 (−1)	NA
	2015-2016	62 046 416	−5 285 848 (−9)	−3 670 902 (69)	−2 021 256 (38)	451 782 (−9)	−45 475 (1)
	2016-2017	56 760 568	−12 544 900 (−22)	−7 221 489 (58)	−2 373 834 (19)	35 579 (0)	−2 985 163 (24)
	2017-2018	44 215 668	−7 813 388 (−18)	−3 519 552 (45)	−2 613 501 (33)	195 299 (−2)	−1 875 636 (24)
	2018-2019	36 402 280	−4 727 454 (−13)	−950 260 (20)	−1 885 833 (40)	−455 354 (10)	−1 436 007 (30)
Infliximab	2013-2014	582 304 960	20 926 912 (4)	−10 802 447 (−52)	NA	31 729 376 (152)	NA
	2014-2015	603 231 872	25 605 056 (4)	−13 701 386 (−54)	NA	39 306 452 (154)	NA
	2015-2016	628 836 928	33 397 056 (5)	−9 895 122 (−30)	−1032 (0)	43 293 224 (130)	NA
	2016-2017	662 233 984	7 165 632 (1)	−21 415 858 (−299)	92 142 (1)	28 487 878 (398)	1443 (0)
	2017-2018	669 399 616	−46 457 088 (−7)	−22 165 778 (48)	−4 903 120 (11)	−15 859 509 (34)	−3 528 745 (8)
	2018-2019	622 942 528	−100 403 360 (−16)	6 972 860 (−7)	−2 551 065 (3)	−98 249 936 (98)	−6 575 154 (7)
Pegfilgrastim	2013-2014	489 723 168	8 185 216 (2)	−43 194 280 (−528)	NA	51 379 480 (628)	NA
	2014-2015	497 908 384	19 603 296 (4)	−24 055 836 (−123)	NA	43 659 148 (223)	NA
	2015-2016	517 511 680	25 767 872 (5)	−20 475 644 (−79)	NA	46 243 532 (179)	NA
	2016-2017	543 279 552	−23 367 712 (−4)	−61 498 924 (263)	NA	38 131 208 (−163)	NA
	2017-2018	519 911 840	−2 996 448 (−1)	−47 701 276 (1592)	−270 029 (9)	44 974 860 (−1501)	NA
	2018-2019	516 915 392	−7 860 928 (−2)	356 310 (−5)	−6 759 196 (86)	−872 689 (11)	−585 399 (7)
Epoetin alfa	2013-2014	187 711 280	−8 390 384 (−4)	−21 036 362 (251)	NA	12 645 990 (−151)	NA
	2014-2015	179 320 896	−11 651 936 (−6)	−21 942 660 (188)	NA	10 290 716 (−88)	NA
	2015-2016	167 668 960	−2 992 064 (−2)	−7 497 195 (251)	NA	4 505 130 (−151)	NA
	2016-2017	164 676 896	−3 600 672 (−2)	−12 696 600 (353)	NA	9 095 931 (−253)	NA
	2017-2018	161 076 224	−18 313 072 (−11)	−11 751 375 (64)	−16 209 (0)	−6 545 483 (36)	NA
	2018-2019	142 763 152	−21 059 832 (−15)	−6 839 935 (32)	−2 218 750 (11)	−11 982 757 (57)	−18 391 (0)

Abbreviation: NA, not applicable.

^a^ Annual total Medicare Part B spending and administration units come from the Centers for Medicare & Medicaid Services’ Part B National Summary Data Files. Units were converted to standard doses (filgrastim, 1 μg; infliximab, 10 mg; epoetin alfa, 1000 units; and pegfilgrastim, 6 mg). Total spending and standard doses were used to calculate Medicare observed prices. The decomposition method followed the Congressional Budget Office’s total physician spending decomposition (eMethods in the Supplement).^5^

## Discussion

There is controversy over whether biosimilars effectively create savings for US payers.^6^ In this cross-sectional study, we found initially modest, though growing, Medicare spending declines after biosimilar launches. However, a limitation of this study is that biosimilar launches are still too recent to know if these declines will persist. Declines in Medicare spending after biosimilar entry came from a range of sources: biosimilar prices and market share drove filgrastim declines, originator prices drove infliximab and epoetin alfa declines, and biosimilar market share drove pegfilgrastim declines. Originator products that treat chronic conditions appear to decrease prices to maintain market share, whereas filgrastim, an acute-condition medication, maintained prices. Future work should evaluate these trends in more recent biosimilar launches.

## Notes

Supplement. eMethods.
