# Supplementary material for: Changes in Medicare Part B Spending for Biologic Drugs After Biosimilar Entry Into the Market
Source: JAMA Health Forum. 2021 Sep 17;2(9):e212634. doi: 10.1001/jamahealthforum.2021.2634 (PMC8796923; doi:10.1001/jamahealthforum.2021.2634)
Supplement: Supplement. — eMethods. [file jamahealthforum-e212634-s001.pdf]

Supplemental Online Content

Dean EB, Bond AM. Changes in Medicare Part B spending for biologic drugs after biosimilar entry into the market. *JAMA Health Forum*. 2021;2(9):e212634. doi:10.1001/jamahealthforum.2021.2634

eMethods

This supplemental material has been provided by the authors to give readers additional information about their work.

© 2021 Dean EB et al. *JAMA Health Forum.*

eMethods

Decomposition method

Decomposition of total spending changes followed the Congressional Budget's Office method of decomposing total physician spending.[1] Their method decomposed prices, *P*, and volume, *V*, using the equation below:

$$
\begin{aligned}
P^{t+1}V^{t+1} - P^tV^t &= (P^{t+1}V^{t+1} - P^{t+1}V^t) + (P^{t+1}V^t - P^tV^t) \\
&= P^{t+1}(V^{t+1} - V^t) + (\mathrm{P}^{\mathrm{t+1}} - P^t)V^t \\
&= P^{t+1}\Delta V + \Delta P V^t
\end{aligned}
$$

We extended this method to 1) separate originator and biosimilar prices, P_o and P_b, and 2) separate overall volume from shifting volume from originator to biosimilar (that is, biosimilar market share or *MS*). Our decomposition methods is represented by the equation below:

$$
\begin{aligned}
&[P_b^{t+1} * MS^{t+1} * V^{t+1} + P_o^{t+1} * (1 - MS^{t+1}) * V^{t+1}] - [P_b^t * MS^t * V^t + P_o^t * (1 - MS^t) * V^t] \\
&= \Delta P_b * MS^t * V^t + \Delta P_o * (1 - MS^t) * V^t \ (a) \\
&+ P_b^{t+1} * \Delta MS * V^t + P_o^{t+1} * -\Delta MS * V^t \ (b) \\
&+ P_b^{t+1} * MS^{t+1} * \Delta V + P_o^{t+1} * (1 - MS^{t+1}) * \Delta V \ (c)
\end{aligned}
$$

Where (a) is the change in total spending due to biosimilar price and originator price changes, (b) is the change in total spending due to biosimilar market share changes and (c) is the change in total spending due to total volume changes.

Note, we defined overall volume as standardized administration volume across originator and biosimilars. We defined biosimilar market share as the standardized volume of biosimilars divided by the overall standardized volume.

[1] Congressional Budget Office. Factors underlying the growth in Medicare's spending for physicians' services [Internet]. Washington (DC): CBO; 2007 Jun [cited 2021 Apr 2]. Available from: https://www.cbo.gov/sites/default/files/110th-congress-2007-2008/reports/06-06-medicarespending.pdf

© 2021 Dean EB et al. *JAMA Health Forum.*

Four products and their competition

Product class	Products within class (reference product italicized)	HCPCs	FDA approval	US market entry	Dosage
filgrastim	*filgrastim (Neupogen)*	J1440<br>J1441<br>J1446			300 MCG<br>480 MCG<br>1 MCG
	filgrastim-sndz (Zarxio)	Q5101	Mar-15	Sep-15	1 MCG
	filgrastim-aafi (Nivestym)	Q5110	Jul-18	Oct-18	1 MCG
	tbo-filgrastim (Granix)*	J1447	Aug-12	Nov-13	5 MCG
infliximab	infliximab (Remicade)	J1745			10 MG
	infiximab-dyyb (Inflectra)**	Q5102<br>Q5103	Apr-16	Nov-16	10 MG
	infiximab-abda (Renflexis)	Q5104	May-17	Sep-17	10MG
epoetin alfa	*epoetin alfa (Epogen/Procrit)*	J0885<br>J0886<br>Q4081			1000 Units<br>1000 Units<br>100 Units
	epoetin alfa-epbx (Retacrit)	Q5105<br>Q5106	May-18	Sep-18	100 Units<br>1000 Units
pegfilgrastim	*pegfilgrastim (Neulasta)*	J2505			6 MG
	pegfilgrastim-jmdb (Fulphila)	Q5108	Jun-18	Jul-18	0.5 MG
	pegfilgrastim-cbqv (Udenyca)	Q5111	Nov-18	Jan-19	0.5 MG

* We did not consider tbo-filgrastim as a biosimilar in this study as it was approved through the traditional FDA approval process (351(a)) rather than through the Biologics Price Competition and Innovation Act of 2009 (BPCIA/351(k)).

** Q5102 (description: injection, infliximab, biosimilar, 10mg) was the original billing code for all biosimilar forms of infliximab. In April 1, 2018, the Q5102 code was discontinued, and replaced with the Q5103 and Q5104 codes.

© 2021 Dean EB et al. *JAMA Health Forum.*
